# Clinical, imaging, and biomarker evidence of amyloid- and tau-related neurodegeneration in late-onset epilepsy of unknown etiology

**DOI:** 10.3389/fneur.2023.1241638

**Published:** 2023-09-27

**Authors:** L. Brian Hickman, John M. Stern, Daniel H. S. Silverman, Noriko Salamon, Keith Vossel

**Affiliations:** ^1^Mary S. Easton Center for Alzheimer’s Research and Care, Department of Neurology, David Geffen School of Medicine, University of California, Los Angeles, Los Angeles, CA, United States; ^2^Department of Neurology, UCLA Seizure Disorder Center, David Geffen School of Medicine, University of California, Los Angeles, Los Angeles, CA, United States; ^3^Ahmanson Translational Imaging Division, Department of Molecular and Medical Pharmacology, David Geffen School of Medicine, University of California, Los Angeles, Los Angeles, CA, United States; ^4^Department of Radiological Sciences, David Geffen School of Medicine, University of California, Los Angeles, Los Angeles, CA, United States

**Keywords:** late onset epilepsy of unknown etiology, late onset epilepsy, Alzheimer dementia, epileptic prodromal Alzheimer disease, epileptic preclinical Alzheimer disease, late-onset amyloid Beta-related epilepsy, amyloid, tau

## Abstract

Accumulating evidence suggests amyloid and tau-related neurodegeneration may play a role in development of late-onset epilepsy of unknown etiology (LOEU). In this article, we review recent evidence that epilepsy may be an initial manifestation of an amyloidopathy or tauopathy that precedes development of Alzheimer’s disease (AD). Patients with LOEU demonstrate an increased risk of cognitive decline, and patients with AD have increased prevalence of preceding epilepsy. Moreover, investigations of LOEU that use CSF biomarkers and imaging techniques have identified preclinical neurodegeneration with evidence of amyloid and tau deposition. Overall, findings to date suggest a relationship between acquired, non-lesional late-onset epilepsy and amyloid and tau-related neurodegeneration, which supports that preclinical or prodromal AD is a distinct etiology of late-onset epilepsy. We propose criteria for assessing elevated risk of developing dementia in patients with late-onset epilepsy utilizing clinical features, available imaging techniques, and biomarker measurements. Further research is needed to validate these criteria and assess optimal treatment strategies for patients with probable epileptic preclinical AD and epileptic prodromal AD.

## 1. Introduction

Epilepsy incidence increases with age, with the highest incidence occurring in older adults at almost double the rate observed in young adults (1). A majority of older adults with acquired epilepsy have an underlying cerebrovascular, neoplastic, or other cerebral lesion known to produce seizures (2). However, 25 to 50% of these patients do not have an identifiable etiology of their epilepsy after clinical evaluation and imaging (1, 3–5). This has been named late-onset epilepsy of unknown etiology (LOEU) (6).

Recent research suggests a link between neurodegenerative processes and LOEU. Specifically, amyloid-and tau-related neurodegeneration may contribute to development of some cases of LOEU. Patients with mild cognitive impairment and Alzheimer’s disease (AD) have an increased risk of epilepsy, with lifetime seizure risk of up to 20–64% in patients with AD (7). Seizures were previously thought to primarily occur late in the disease course, but it is now recognized that both clinical and subclinical seizures often occur in early stages of AD as well (8, 9). Animal models of amyloidopathy also consistently demonstrate increased frequency of seizures and epileptiform activity (10–12). Similarly, suppression of amyloid-beta precursor protein in animal models reduces epileptiform activity (13) and reduction in endogenous tau confers resistance to induced seizures (14, 15). In addition to direct pro-epileptogenic effects of amyloid and tau pathology, astrocyte-mediated neuroinflammation has been implicated in both preclinical AD and epilepsy and may be a common mechanism of pathogenesis (16, 17). Seizures promoted by neurodegeneration may in turn contribute to further aggregation of amyloid and tau, leading to further cognitive decline (18–20).

Some instances of LOEU may represent a prodrome of AD, with seizures acting as an early marker for impending cognitive decline. Accumulating evidence from epidemiological, neuroimaging, and biomarker investigations of LOEU strengthens this hypothesis. While LOEU likely does not represent a single, homogenous entity, these studies suggest that prodromal AD may produce late-onset epilepsy and can be identified using clinical and biomarker features. This raises the possibility of improving prognostication and providing a potential early window for intervention. In this review, we discuss the evidence that a subset of patients presenting with late-onset epilepsy have prodromal AD, and we propose a classification scheme for use by researchers that, following validation, can be considered for clinical use.

## 2. Evaluation of patients with late-onset epilepsy

**Figure 1:**
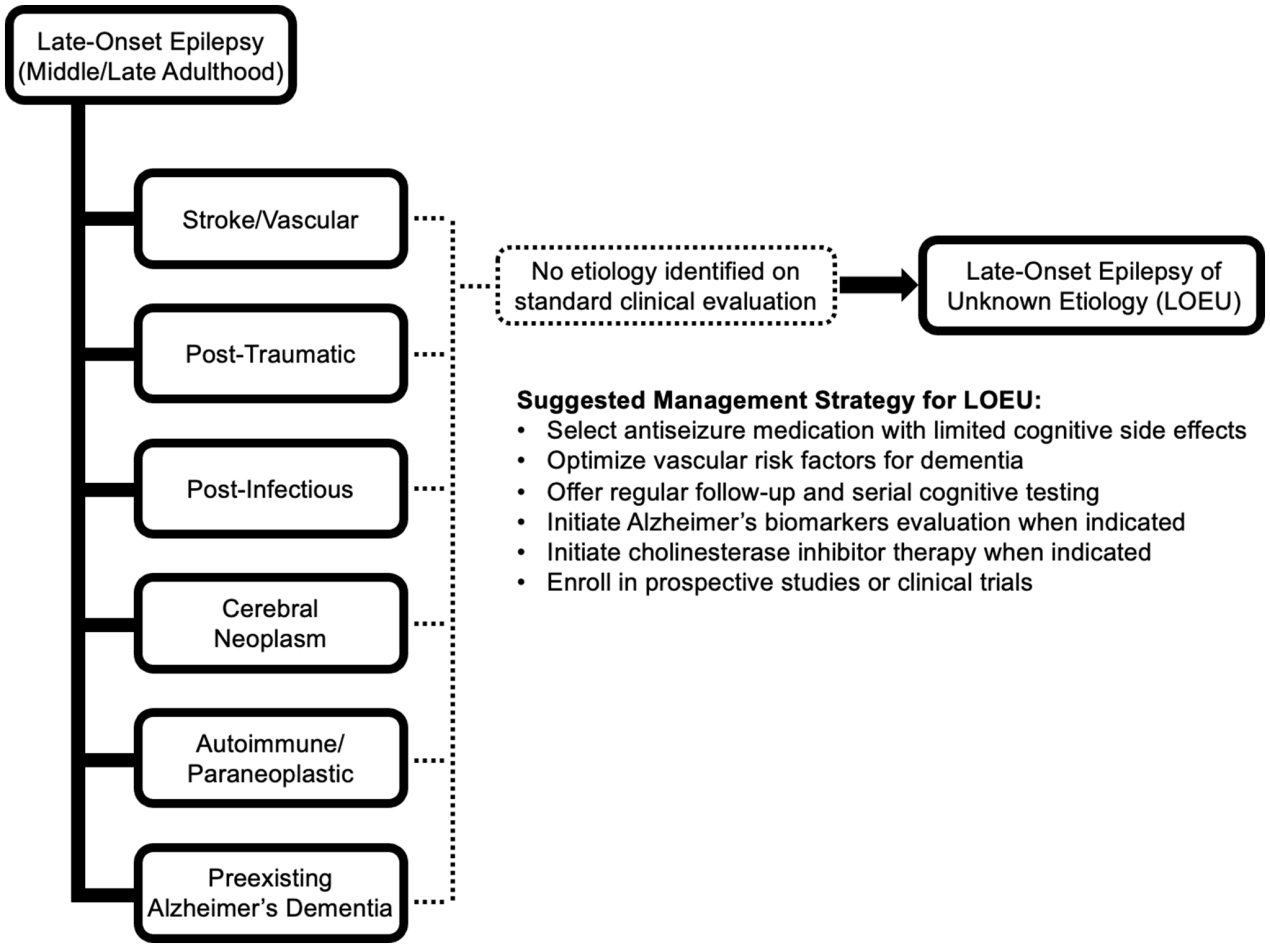
Potential etiologies of late-onset epilepsy. If no etiology can be identified after comprehensive standard clinical evaluation including EEG, epilepsy-protocol MRI, and detailed clinical assessment, the presentation is consistent with late-onset epilepsy of unknown etiology (LOEU). Management of LOEU mirrors general management of epilepsy in older adults, with greater emphasis on monitoring for development of cognitive impairment and intervening on comorbid risk factors for cognitive decline.

Standard literature definitions of late-onset epilepsy of unknown etiology specifies an age of onset cutoff at age 55 or older, without prior history of seizures earlier in life, though some studies use age cutoffs ranging between 40 and 65 (21–23). The evaluation of new-onset epilepsy includes a thorough clinical assessment with detailed neuroimaging, toxic/metabolic laboratory evaluation, and an electroencephalogram (EEG) (24). Aside from an age cutoff, LOEU is otherwise only defined by absence of a clear etiology for developing epilepsy despite completing a standard, comprehensive clinical evaluation. Common causes of acquired, late-onset epilepsy that are important to exclude include ischemic or hemorrhagic cerebral cortical infarction, tumor, post-traumatic encephalomalacia, and preexisting neurodegeneration (Figure 1). Less commonly, late-onset epilepsy can be caused by prior cerebral infection or autoimmune/paraneoplastic disease, and evaluation should be tailored to include cerebrospinal fluid analysis and autoantibody testing when imaging and clinical features suggest an inflammatory process (25).

Given that patients may have structural lesions that are clinically silent aside from producing seizures, high-resolution neuroimaging is an essential tool for evaluating presence of possible structural etiologies. Determination of LOEU should only be made after careful expert review of an epilepsy protocol MRI, preferably obtained using a 3 T MRI machine and including sequences and slice thicknesses recommended by the ILAE Neuroimaging task force for evaluation of epilepsy (26). A detailed susceptibility weighted imaging sequence or gradient echo sequence should also be obtained to detect potentially explanatory cortical microhemorrhages. Though global or focal atrophy is commonly observed, LOEU is defined as “MRI-negative” epilepsy, signifying absence of an explanatory lesion while allowing likely incidental findings. Presence of a lesion likely to produce epilepsy essentially excludes LOEU. However, more reliable determination that a cerebral cortical lesion is unrelated would typically require detailed ictal video-EEG recordings or intracranial EEG recordings, which is not often clinically justifiable, especially when seizure control has been achieved.

Currently, it is unclear whether MRI evidence of hippocampal sclerosis reliably indicates an underlying etiology in LOEU because hippocampal sclerosis is more commonly an etiology of epilepsy with onset earlier in life. Hippocampal sclerosis is less clearly a distinct etiology in late-onset epilepsy, as hippocampal sclerosis in older adults may be produced by multiple pathologic processes, including ischemic injury, AD, and other TDP-43 related diseases. Overall, the presence of hippocampal sclerosis on imaging in LOEU likely does not shed light on a single, unifying etiology (27–29). The difficulty in clinically interpreting hippocampal sclerosis in the older adult has complicated LOEU research, with some studies of LOEU excluding patients with imaging findings of hippocampal sclerosis (30) while others including frank atrophy and sclerosis (21, 31). Further work is needed to assess temporal evolution of hippocampal atrophy and sclerosis in LOEU or if particular imaging or pathologic features of hippocampal sclerosis in LOEU can be connected with specific probable etiologies.

## 3. Common clinical findings in patients with late-onset epilepsy of unknown etiology

Features of seizures in LOEU are largely consistent and are predominantly focal in manifestation. These may be focal with impaired awareness or focal with progression to bilateral tonic–clonic seizures. Focal with intact awareness (an aura in isolation) also may occur. Usually, 15% or fewer patients with LOEU are described as having generalized seizures (32–35). While evaluated as generalized, many of these may be focal onset based on observations that the pathophysiological process producing late-onset epilepsy rarely result in new onset generalized seizures, which typically have onset in childhood to adolescence.

In keeping with a focal onset, the most common EEG abnormalities described in LOEU are focal epileptiform discharges and focal slowing (22, 33, 35). The typical location for epileptiform discharges or slowing is in either unilateral or bilateral temporal lobes. EEG recordings without evidence of epileptiform abnormalities or focal slowing are common, but this may relate to the EEG recording conditions. EEG sensitivity is increased by longer duration of recordings, repeated recordings, and recordings that include sleep. Older adult patients may undergo a limited duration of EEG recording because of lower seizure frequency and rate of medication resistance compared to younger patients. Recordings during sleep significantly increase yield for epileptic abnormalities in LOEU (21) Publications on LOEU rarely describe EEG recording details.

While age 55 is typically used as the age cutoff for categorizing LOEU, the average age for onset of LOEU is often reported to be between 60 and 70 years (32, 35, 36). Patients with LOEU and preexisting mild cognitive impairment (MCI) are older on average than patients with LOEU and without preexisting MCI (35). A large majority of patients with LOEU are reported to respond to initial antiseizure medication treatment and rarely require polytherapy (6, 32–34). Evaluation for surgical treatment due to lack of medication responsiveness is uncommon.

## 4. Epidemiological evidence of an association between LOEU and AD

The most substantial evidence that some cases of LOEU may be a manifestation of preclinical or prodromal AD comes from retrospective epidemiological studies. Separate epidemiological literature on both AD and LOEU support an association (Table 1).

**Table 1:** Epidemiological studies describing an association between LOEU and AD/aMCI.

Investigations of LOEU				
Authors	Year of Publication	Research Design	Average age of epilepsy onset (years)	% LOEU developing dementia
Ophir et al.	2021	Retrospective	61	22%
Kawakami et al.	2018	Retrospective	Not reported	21%
Keret et al.	2020	Retrospective	Not reported	8.3%
Costa et al.	2019	Prospective	Not reported	25%
Johnson et al.	2020	Prospective	Not reported (67 or older)	41.6%
Investigations of AD/aMCI				
Authors	Year of Publication	Research Design	Average age of epilepsy onset (years)	% AD/aMCI with preceding epilepsy
Samson et al.	1996	Retrospective	Not reported	7%
DiFrancesco et al.	2017	Retrospective	68	1.7%
Vossel et al.	2013	Retrospective	68	3.1%
Sarkis et al.	2016	Retrospective	74	2.3%
Cretin et al.	2016	Retrospective	63	3.1%

Patients diagnosed with AD have an elevated rate of seizures in the years prior to their diagnosis. In an investigation of patients with early-onset AD (onset before age 65), Samson et al. (37) found that 7% had a history of seizures occurring before AD diagnosis. Similarly, DiFrancesco et al. (38) found that patients with AD had a 17-fold increased risk of preceding LOEU compared to a reference population. AD patients with prior LOEU had onset of epilepsy an average of 4.5 years before AD diagnosis. Vossel et al. (8) investigated patients with diagnosis of AD/aMCI and epilepsy; 83% of patients had onset of epilepsy either preceding or occurring near time of diagnosis, and in 38%, seizures preceded or coincided with onset of cognitive decline. Other studies have confirmed that LOEU may occur before initial cognitive decline in patients who develop AD or MCI. Sarkis et al. (39) investigated patients with both dementia and MRI-negative epilepsy, and found that 8% had onset of epilepsy before documented cognitive decline and 25% had onset prior to a diagnosis of dementia. While this investigation was not limited to AD and did not include subgroup analyses, over 80% of the sample had possible, probable, or autopsy-proven AD. Cretin et al. (32) retrospectively investigated patients at an academic medical center who met criteria for MCI and found that 3.1% of patients with MCI and 5% of patients with amnestic MCI had epilepsy without a defined etiology preceding cognitive complaints. All patients with MCI and preceding LOEU had amnestic MCI, which is associated with an elevated risk of progression to AD (40). Cretin et al. (32) found that these patients developed seizures an average of 2.7 years before self-reported cognitive decline and 6.9 years before a diagnosis of MCI.

Retrospective investigations of LOEU have also identified a subsequent increased risk of developing dementia. Ophir et al. (36) investigated patients with LOEU initially presenting without cognitive symptoms at ages between 55 and 69 found a 10-year cumulative incidence of dementia of 22.2%; mortality at 10 years in this sample was 31%, potentially preventing additional patients from expressing an eventual dementia. Incidence of dementia was higher among patients with LOEU and temporal discharges on baseline EEG, with 10 of 17 developing dementia during the retrospective study period. Kawakami et al. (22) found that patients with LOEU had a 21% cumulative incidence of dementia after 5 years follow-up compared to 4.3% of controls. Utilizing a random sample of patients from the Veterans Health Database, Keret et al. (41) studied patients with onset of epilepsy at age 55 or above who lacked an ICD-9-CM code to explain the cause of their epilepsy. After an average 6.1 years of follow-up, these patients had a hazard ratio of 1.89 (95% CIL 1.62–2.20) for a diagnosis of dementia compared to patients above age 55 who did not develop epilepsy. These investigations did not specifically determine if patients who developed dementia had probable AD versus another etiology.

There are few published prospective studies that follow patients with newly diagnosed LOEU to assess the rate of AD diagnosis. Costa et al. (33) describes prospective follow up of patients with LOEU who were cognitively normal at initial evaluation for up to 5 years. Of these patients, 25% developed dementia with 17.5% meeting criteria for AD during the follow up period. In a prospective study of patients with epilepsy starting at age 67 or later, Johnson et al. (42) found that late-onset epilepsy was associated with an increased risk of subsequent diagnosis of dementia, with an adjusted hazard ratio of 3.05 (95% CI, 2.65–3.51). Median time from diagnosis of epilepsy to diagnosis of dementia was 3.7 years. While this investigation did not solely include patients with LOEU, patients without a history of stroke had an even greater risk of subsequent dementia, with a hazard ratio of 3.39 (95% CI, 2.89–3.97).

Epidemiological associations demonstrate an association between LEOU and increased risk of subsequent stroke (43) and vascular risk factors increase risk of developing late-onset epilepsy (44). This suggests that LOEU is not be a homogenous entity and that previously undetected cerebrovascular disease may be an underlying etiology in some patients. Alternatively, this may reflect shared underlying mechanisms contributing to cerebrovascular disease, AD, and epilepsy.

Existing publications rarely compare risk of cognitive decline in LOEU patients with late-onset epilepsy of known etiology or patients with early-onset epilepsy. Patients with late-onset epilepsy in general demonstrate increased risk of developing dementia (45) potentially related to increased risk of developing vascular dementia in patients with cerebrovascular disease as a cause of epilepsy (46). Epilepsy itself is associated with increased risk of dementia and amyloid pathology (47), even when first diagnosed in early life. In addition to direct structural and functional effects from seizures themselves, potential factors that may impair cognition after development of epilepsy can include traumatic brain injuries from seizure-related accidents (48), medications used to treat epilepsy (49, 50), and epilepsy surgeries (51). Thus, dedicated prospective investigations comparing cognitive decline across all etiologies and ages of onset of epilepsy are warranted to fully identify which patients are at greatest risk of developing AD. As seizure burden is typically low in LOEU and risk of progression to AD appears to be higher specifically in LOEU than in patients with other forms of epilepsy (33, 36, 52), it is likely that the occurrence of seizures in LOEU is reflective of underlying AD pathology, rather than seizures being the major driver of AD onset.

## 5. Quantitative cognitive testing in LOEU

In addition to epidemiological associations between LOEU and development of AD, quantitative cognitive testing has shown impaired cognitive performance in patients with LOEU compared to controls. Fernandes et al. (34) studied patients with LOEU with a Mini-Mental State Examination (MMSE) score greater than 24 and found that patients with LOEU had globally lower cognitive testing performances, including on tests of recall (Rey Auditory Verbal Learning Test), verbal fluency (Phonological Verbal Fluency test, Semantic verbal fluency test), and executive function (Rey–Osterrieth Complex Figure Test) compared to controls. After 12 months, patients with LOEU showed progressive impairment in the memory domain with lower RALVT-I scores, while controls showed memory improvement with an increase in RAVLT-I scores. Ligori et al. (30) studying patients with LOEU with cutoff MMSE scores above 24 found that patients with LOEU had a statistically significant decline in MMSE scores and word recall at 12 months follow up, though the average decline was small (less than 1 point on average for both tests) and scores on phonological verbal fluency increased. Differences in cognitive score changes between different antiseizure medication regimens were small.

Initial impairment in quantitative cognitive testing is not universally seen; Costa et al. (6, 33) investigated patients with LOEU without dementia and demonstrated no difference in MMSE scores between LOEU and healthy controls at time of enrollment, despite a later 20% rate of progression to dementia after 3 years. Similarly, Nardi Cesarini et al. (35) did not find differences between cognitively normal patients with LOEU and healthy controls. However, patients with LOEU and comorbid MCI had lower average MMSE scores, clock drawing scores, phonemic/letter fluency, and abstract logical reasoning scores than age-matched patients with MCI without epilepsy.

## 6. Genetic markers that confer increased risk of late-onset epilepsy and AD

While genetic investigations have not specifically evaluated LOEU, recent studies have evaluated shared genetic risk factors between late-onset epilepsy and AD. APO-ε4 is a known genetic risk factor for amyloid pathology and AD (53). As part of a prospective cohort study, Johnson et al. (44) found that carrying an APO-ε4 allele was associated with increased risk of developing late-onset epilepsy. This also demonstrated a dose-dependent relationship, with patients carrying two APO-ε4 alleles demonstrating an adjusted hazard ratio of 2.36 (95% CI: 1.65–3.38) and patients carrying a single APO-ε4 demonstrating an adjusted hazard ratio of 1.42 (95% CI: 1.19–1.69). Results held when excluding patients who were diagnosed with stroke or dementia. A subsequent study using the same sample but accounting for additional follow-up time again demonstrated an increased risk of late-onset epilepsy in patients with two APO-ε4 alleles (42). Using a mendelian randomization analysis, Fang et al. (54) found that a genome-wide genetic predisposition to AD was associated with a small but significantly increased risk of both focal epilepsy with hippocampal sclerosis (OR 1.01, 95% CI: 1.004–1.022) and generalized epilepsy (OR 1.05, 95% CI: 1.003–1.105). Genetic predisposition to focal epilepsy with hippocampal sclerosis was also found to be associated with increased risk of AD (OR 3.99).

## 7. Cerebrospinal fluid biomarker evidence of AD pathophysiology in LOEU

With the advent of precision CSF biomarkers and imaging modalities for detecting amyloid and tau pathology, there has been a concerted effort to develop a biomarker-based classification framework for diagnosis of AD. CSF biomarkers are well validated for early, antemortem detection of pathological findings in patients with MCI and AD (55). A NINDS-supported biomarker and neuroimaging-based diagnostic framework utilizes these biomarkers in order to achieve improved diagnostic accuracy, reduce antemortem misdiagnoses, and improve prognostic accuracy of cognitive trajectory (56). This framework is termed the AT(N) classification system, referring to the presence of amyloid, tau, and neurodegeneration in pathologically definite AD. Reduced levels of CSF Aβ_1-42_ or reduced ratio of Aβ_1-42_ to Aβ_1-40_ are indicative of amyloid pathology, including amyloid plaques (56). Increased levels of CSF phosphorylated tau (p-tau) indicate presence of pathologic tau neurofibrillary tangles and increased levels of CSF total tau (t-tau) are correlated with greater neuronal loss and neurodegeneration.

Investigations of patients with LOEU using Aβ_1-42_ and p-tau CSF biomarkers suggest that some patients have a previously unrecognized amyloidopathy and/or tauopathy. Cretin et al. (32) reported a series of patients with MCI and preceding LOEU. All 13 patients demonstrated either low CSF Aβ_1-42_ (53.8%) or low CSF Aβ_1-42_/Aβ_1-40_ ratio (46.2%) by the time of MCI diagnosis. Average p-tau levels were found to be elevated. Costa et al. (6, 33) investigated patients with LOEU with MMSE scores greater than 24 and found that 37.5% of patients with LOEU had CSF Aβ_1-42_ below cutoff pathological levels. As a group, patients with LOEU had lower CSF Aβ_1-42_ compared to healthy controls, despite similar cognitive performance on tests of recall, attention, and executive function. Patients with LOEU also had significantly greater t-tau levels in CSF, though p-tau levels were not significantly different compared to controls. Importantly, 6 of the 15 patients with LOEU and positive Aβ_1-42_ biomarker levels developed a clinical dementia and 5 met clinical criteria for AD during an average 3 year follow up period. In contrast, 4 out of 25 patients with LOEU without positive Aβ_1-42_ biomarker levels developed dementia during the follow up period, 2 of whom met criteria for AD. Fernandes et al. (34) also studied LOEU patients without preexisting diagnosis of MCI and with MMSE greater than 24. Investigators found lower CSF Aβ_1-42_ and both higher CSF p-tau and t-tau levels compared to controls. Of the sample of 55 patients, 16.4% met cutoff levels of pathologically low CSF Aβ_1-42_.

Decreased Aβ_1-42_ is not uniformly observed in LOEU without preexisting MCI. In an investigation by Nardi Cesarini et al. (35) of patients with LOEU with or without comorbid MCI, patients with LOEU without comorbid MCI did not have a significant difference in Aβ_1-42_ levels compared to controls. Patients with both LOEU and MCI had lower average CSF Aβ_1-42_ and Aβ_1-42_/p-tau compared to LOEU without MCI, with 41% demonstrating pathologically low levels. 22.7% of MCI-LOEU had both pathologically decreased amyloid and increased p-tau, meeting both (A+/T+) classification. None of the cognitively normal LOEU patients had positive CSF (A) or (T) classification.

## 8. Imaging findings suggestive of AD pathophysiology in LOEU

By definition, patients with LOEU lack visible structural abnormalities on imaging that are known to produce epilepsy, but visual assessment and volumetric analyses often demonstrate findings suggestive of imaging abnormalities seen in both early AD and occult cerebrovascular disease. Using visual inspection, Nagino et al. (57) found that 58% of patients with LOEU had global atrophy on MRI and 48% had unilateral or bilateral hippocampal atrophy. The most common imaging abnormality was white matter hyperintensities (a feature of cerebrovascular disease), which was present in 81% of the sample. In a sample of 66 patients with LOEU, Sarkis et al. (21) found that 81% had evidence of temporal atrophy and 21% had moderate or severe hippocampal volume loss assessed using a visual inspection scale validated to predict progression to MCI and AD. In this sample, 34.8% had small-to-large confluent white matter hyperintensities based on visual inspection. In Cretin et al.’s^32^ investigation of patients with MCI with preceding LOEU, 12 out of 13 patients demonstrated mild bilateral hippocampal atrophy on visual inspection. In the same study, over two-thirds also had cerebrovascular white matter lesions and over one-third had subcortical lacunes or non-cortical microhemorrhages.

Using quantitative analyses, Hanby et al. (23) also found that patients with LOEU had lower global cortical volume than age-matched controls and had increased burden of white matter hyperintensities. Johnson et al. (58) found that lower total cortical volume was associated with increased likelihood of late-onset epilepsy; while this investigation did not specifically identify patients with LOEU, results held when excluding patients with a diagnosis of stroke or pre-existing dementia. Kaestner et al. (31) performed a detailed investigation of quantitative MRI measures in 23 patients with late-onset TLE, defined in this investigation as patients with onset of TLE after age 50. Compared to healthy controls, patients with late-onset TLE had prominent cortical thinning in mesial temporal lobes, lateral temporal lobes, prefrontal, precentral, and paracentral regions. Directly comparing patients with late-onset TLE to patients with early-onset TLE, patients with LO-TLE had thinner cortex in bilateral fusiform gyri. Of note, this difference in cortical thickness was found even though patients with EO-TLE had approximately 30 years greater duration of epilepsy compared to patients with LO-TLE. Patients with EO-TLE were more likely to have hippocampal sclerosis (58% versus 26%) as assessed by visual inspection.

In addition to MRI evidence of neurodegeneration, FDG-PET also demonstrates evidence of changes suggestive of AD. Using FDG-PET scans, Fernandes et al. (34) found that patients with LOEU had significantly reduced glucose metabolism in the right posterior cingulate cortex and left precuneus compared to controls. Decreased glucose in these regions was correlated with worse recall on both the immediate and delayed Rey Auditory Verbal Learning Test. DiFrancesco et al. (59) also investigated patients using cerebral FDG-PET, revealing temporal lobe hypometabolism in 87% of patients with LOEU. While five patients had multifocal decreased metabolism in temporal lobe structures, the other 15 patients had focal locations of temporal hypometabolism, most commonly in the anterior temporal lobe. Cases without hypometabolism in the temporal lobe had focal hypometabolism in the caudate nucleus. Nearly all patients with lateralized focal slowing or epileptiform discharges on EEG had congruent laterality of hypometabolism.

There have been few investigations using of amyloid or tau specific PET scans to study presence of amyloidopathy and tauopathy in patients with LOEU. Sarkis et al. (60) investigated six patients, ages 69–83, with history of nonlesional epilepsy and cognitive decline consistent with MCI or early dementia using F-18 florbetaben amyloid PET scans. Five of the six patients had seizures preceding cognitive decline; the sixth patient had onset of seizures 2 years following onset of cognitive decline. Four of the six patients had positive amyloid scans assessed by visual inspection. These results and results from CSF studies suggest that amyloid and tau-specific PET scans may be a useful method for assessing patterns of amyloid and tau deposition in patients with LOEU progressing to AD.

## 9. Directions for pathologic studies in LOEU

Despite an existing literature on amyloid and tau pathology in a broad array of patients with epilepsy, there is an absence of publications on pathologic analysis of brain tissue from patients specifically with LOEU. This may be due to both low rates of pharmacoresistant seizures, which obviates a need for surgical resection and therefore reduces the possibility for histopathological review. Enrolling patients into longitudinal studies that include eventual cerebral pathological review after death may reveal distinct patterns of neurodegeneration. In particular, tissue comparisons between patients with LOEU who progress to AD and patients who do not, combined with antemortem EEG or MEG-based localizations of seizure onset zones, may demonstrate molecular and histological findings to explain why some patients demonstrate progressive cognitive decline while others do not. Existing AD tissue banks that include patients who had onset of epilepsy prior to cognitive degeneration may demonstrate distinct patterns of neurodegeneration compared to patients with AD who did not have seizures. Patterns of mesial temporal lobe neurodegeneration may be particularly significant to assess in LOEU given early involvement of the temporal lobe in AD and frequency of temporal EEG and mesial temporal imaging abnormalities in LOEU. TDP-43 protein deposition is often observed in AD with prominent hippocampal sclerosis (28); as CSF biomarker measurement of TDP-43 has limited utility, pathological assessment of TDP-43 in addition to amyloid plaques and tau neurofibrillary tangles should be included in LOEU cases.

As animal models of epilepsy suggest that seizures themselves may induce increased amyloid and tau depositions, pathologic comparisons between patients with LOEU and patients with early onset of epilepsy are also needed to further discern what findings may be a driving factor in producing seizures and what findings are expected as a result of seizures. Existing literature on amyloid and tau pathology in early-onset temporal lobe epilepsy has demonstrated inconsistent results. Resected temporal lobe tissue in a study of patients with pharmacoresistant TLE has demonstrated higher rates of amyloid plaques compared to age-matched autopsy controls (61). In another study, resected tissue demonstrated increased neuronal immunoreactivity for amyloid precursor protein, but did not show higher rates of plaques (62). Tai et al. (63) investigated resected temporal lobe tissue from patients with temporal lobe epilepsy. Typical age of onset of epilepsy occurred in the second decade of life with resections occurring between ages 50–65. 94% of the sample demonstrated evidence of tau neuropathology visualized as neuropil threads, neurofibrillary tangles, or pre-tangles, but prevalence of increased Braak stages (limited to temporal lobe assessment) was not significantly higher in TLE patients than age-match controls. In contrast, in a study of post-mortem pathologic analyses from patients with chronic epilepsy showed increased Braak stages in patients aged 40–65 compared to controls in the same age group, with higher average Braak stages noted in patients with focal onset epilepsy compared to generalized onset (64). Braak staging did not correlate with presence of hippocampal sclerosis.

## 10. Classification of LOEU as demonstrating epileptic prodromal AD, epileptic preclinical AD, or late-onset Aβ-related epilepsy

In the AT(N) classification scheme, patients with positive amyloid biomarkers are considered to fall on the AD continuum; the presence of a positive amyloid biomarker study alone confers increased risk of cognitive decline in cognitively normal adults (65). It is currently unclear if, given enough time and absence of other causes of mortality, what percentage of patients with positive amyloid biomarkers would eventually develop AD. Presence of both positive amyloid and tau biomarkers establishes particularly elevated risk of progression to AD in MCI and is termed preclinical AD in cognitively normal individuals (66).

**Figure 2:**
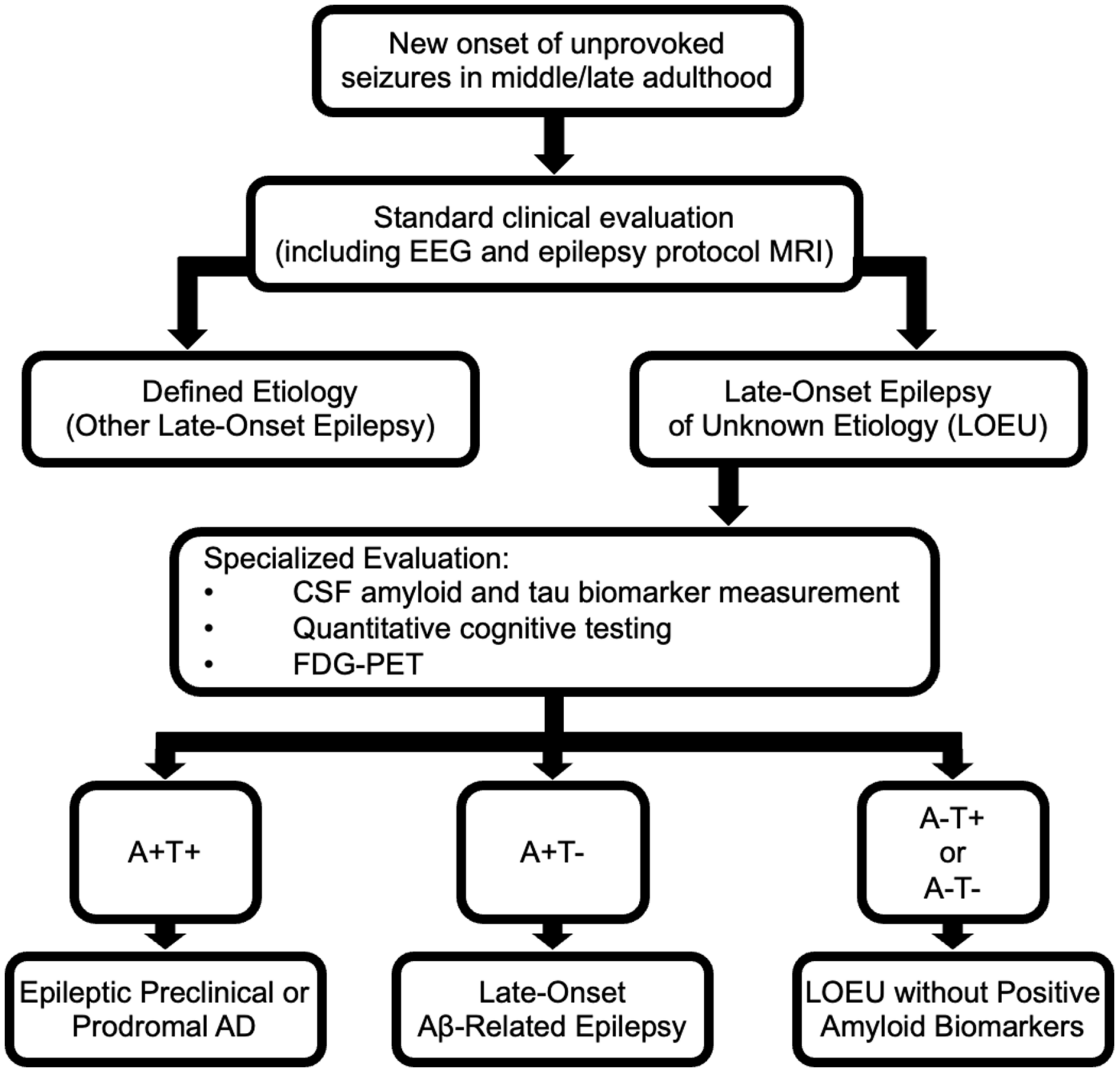
Evaluation of late-onset epilepsy of unknown etiology (LOEU) and classification based upon results of biomarker testing and imaging. The AT(N) classification system for AD can be used to classify patients with late-onset epilepsy. “A” refers to amyloid biomarkers; “T” refers to tau biomarkers indicative of neurofibrillary tangles; “N” refers to evidence of neurodegeneration assessed by neuroimaging or biomarkers. PART, Primary Age-Related Tauopathy.

Given established epidemiological associations between LOEU and AD, imaging abnormalities in LOEU suggestive of neurodegenerative processes, and results of biomarker testing in LOEU, we propose that amyloid and tau biomarkers can be used to further classify patients with LOEU (Figure 2). Presence of both positive amyloid and tau biomarkers in a patient with late-onset epilepsy with otherwise unknown etiology may be sufficient to categorize patients as demonstrating epileptic preclinical AD or epileptic prodromal AD. In cases without MCI and both positive amyloid and tau neurofibrillary tangle biomarkers, we propose categorizing patients as demonstrating “epileptic preclinical AD,” corresponding with terminology proposed for biopathologic preclinical AD (56). In patients with documented MCI, positive amyloid biomarker testing, and positive tau neurofibrillary tangle biomarker testing, we suggest categorization as “epileptic prodromal AD,” a term previously proposed by Cretin et al. (32) and again corresponding with existing biopatholic research terminology. While further prospective studies are needed, the presence of LOEU, positive amyloid biomarker testing, and positive tau biomarker testing may each signify independent risk factors for progressive AD-related cognitive decline leading to dementia. A categorization of epileptic preclinical or prodromal AD is significant as it implies that the etiology of epilepsy is no longer unknown but is strongly suspected to be a result of AD pathology.

As patients with LOEU prior to development of MCI/dementia inconsistently demonstrate positive tau biomarkers, but frequently show positive amyloid biomarkers, an additional classification category is needed for patients with positive amyloid but with negative tau markers. These patients may be considered to demonstrate late-onset Aβ-related epilepsy (LAβE), as previously proposed by Romoli et al. (67) This terminology implies that there is some uncertainty regarding eventual development of frank AD and that underlying etiology of epilepsy is not definite, but with suspicion that an amyloidopathy is playing a contributing role.

Studies of biomarkers in LOEU note infrequent instances of patients with pathologic levels of p-tau in CSF, but non-pathologic Aβ_1-42_ levels (35). CSF biomarker measurements positive for tau pathology but negative for amyloid pathology may seem suggestive of primary age-related tauopathy (PART) (68), but recent research suggests increased soluble CSF p-tau is more closely correlated with amyloid deposition than tau neurofibrillary tangles as measured through PET imaging (69, 70). It is unclear how to classify LOEU with (A−/T+) biomarker profiles at this time. Pathological tau depositions may be a primary driver of seizure activity, occur as a result of seizures stemming from another etiology, or represent an incidental co-occurring process such as from PART. Thus, assessment of patients with LOEU using amyloid and tau PET imaging may clarify (A) and (T) status in patients who demonstrate CSF biomarkers suggestive of (A−/T+). Further research on occurrence rates of preceding seizures in PART may help elucidate a possible relationship between tauopathic processes and epilepsy.

Presence of neurodegeneration on neuroimaging (N+) may be considered supportive of but not necessary for classification of epileptic prodromal AD, epileptic preclinical AD, or LAβE. Supportive patterns of neurodegeneration on MRI or FDG-PET mirror those observed in AD, with features of atrophy or hypometabolism in temporal or parietal structures. It is unclear if substantial burden of white matter disease in LOEU suggests that underlying AD pathology is less likely, or if it reflects common shared factors between AD and cerebrovascular disease. Stratification of patients using novel serum biomarkers of cerebrovascular disease and vascular cognitive impairment may clarify this (71).

The bulk of evidence for proposing these criteria comes from results from CSF amyloid and tau biomarkers in patients with LOEU and from studies of cognitively intact individuals without epilepsy who demonstrate positive biomarkers. Investigations that replicate existing biomarker findings in LOEU and evaluate patients with LOEU longitudinally are needed. While the AT(N) classification scheme for AD also incorporates amyloid and tau PET imaging, there have been few studies utilizing amyloid and tau PET imaging in LOEU. Distinct patterns of deposition may be seen in patients with epileptic preclinical and epileptic prodromal AD that could further understanding why some patients with AD develop seizures at earlier stages of the disease compared to others. Likewise, investigations using plasma-based amyloid and tau biomarkers compared to CSF biomarkers in LOEU have not been systematically performed and are also needed. Normalizing Aβ_1-42_ levels in comparison to Aβ_1-40_ (determined through calculating an Aβ_1-42_/Aβ_1-40_ ratio) has been found to increase sensitivity and specificity for discriminating AD from other dementias (72), and this may be useful for assessing for epileptic preclinical or prodromal AD as well. Neurofilament light chain (NFL) may also prove to be elevated in neurodegenerative processes producing LOEU, though this biomarker may have limited specificity for AD (73) and may be impacted by frequency and duration of seizures (74, 75). Other biomarkers undergoing investigation for detecting early preclinical stages of AD, such as the shedded form of platelet-derived growth factor receptor-b and plasma glial fibrillary acidic protein, may supplement existing biomarkers in detecting epileptic preclinical or prodromal AD (16, 76). Particular seizure types, such as temporal lobe seizures, may occur more frequently in epilepsy occurring from early stages of AD and also warrant further study.

Further research may also elucidate important comorbidities that contribute to the observed relationship between LOEU and AD. In addition to both demonstrating increased small vessel cerebrovascular disease, cerebral amyloid angiopathy may contribute to cortical injury and seizures; serial MRI imaging including GRE or SWI may elucidate if suggestive cortical microhemorrhages or siderosis develop over time in LOEU (77). Subclinical seizures and epileptiform activity during sleep may compromise healthy sleep and may play a role in impaired Aβ processing and clearance (78). Traumatic brain injuries occur at an elevated rate in patients with epilepsy and may contribute to development of AD pathology and increased risk of AD (79, 80). AD and epilepsy may both lead to impairments in the blood–brain barrier, contributing to further neuronal dysfunction (81). Lastly, as neurodegenerative processes frequently co-occur, it will be important to continue investigating if co-occurring TDP-43 and alpha-synuclein deposits impact seizure expression.

Currently, categorizing patients as demonstrating epileptic prodromal AD, epileptic preclinical AD, and LAβE is important to recognize clinically, but requires further research to determine an optimal management approach. With further research, these categorizations will be useful for clinicians when counseling patients about prognosis and assessing eligibility for therapies or clinical trials. Phase 3 studies of amyloid lowering antibody therapies excluded patients with recent history of seizures (82, 83). Thus, patients with LOEU may currently have reduced access to anti-amyloid therapies. As the safety of amyloid lowering therapies has not been well investigated in patients with epilepsy, further clinical trials should be performed, including investigating impact on cognitive symptoms, seizure frequency, and development of amyloid related imaging abnormalities. Given recent trial results that found treatment with donanemab had greater benefit when used in early stages of AD (84), it is possible that patients with epileptic preclinical AD without frank cognitive symptoms may be optimal candidates for treatment with anti-amyloid therapies.

At this time, management of epileptic prodromal AD, epileptic preclinical AD, and LAβE should mirror existing strategies for management of late-onset epilepsy and epilepsy in older adults (Figure 1). Avoidance of antiseizure medications with greater cognitive side effects, such as topiramate, zonisamide, and phenobarbital, may be prudent (85). Divalproex sodium has been associated with accelerated cognitive decline and cerebral atrophy in AD; thus treatment with divalproex and other formulations of valproate may not be preferred (86). As levetiracetam has few drug–drug interactions and treatment with low-dose levetiracetam in patients with detectible epileptiform activity has been found to improve spatial memory and executive function (87), levetiracetam or brivaracetam may be preferred initial treatments. Lamotrigine and lacosamide are also well tolerated in older adults, but evidence that these medications and other sodium channel inhibitors slow cardiac conduction is a consideration in patients with existing cardiac comorbidities (88). As causes of dementia are commonly multifactorial, optimization of cerebrovascular risk factors including hypertension, hyperlipidemia, and diabetes mellitus may slow rates of cognitive decline. Annual or biennial cognitive screening may allow for early detection of progression. Upon meeting criteria for AD, early treatment with anticholinesterase inhibitors may reduce cognitive symptoms. Patients meeting criteria for epileptic prodromal AD, epileptic preclinical AD, or LAβE may eventually be offered enrollment in targeted clinical trials.

## 11. Conclusion

Evidence from recent investigations suggests that LOEU can serve as an early sign of AD. Epidemiological data establish a significant association between LOEU and AD, indicating that individuals with LOEU are at an increased risk of developing AD within years of epilepsy onset. Furthermore, biomarker investigations focusing on amyloid and tau show that biomarker profiles may improve prediction of progression to AD in LOEU patients. Based on this evidence, any older adult presenting with LOEU should have a thorough evaluation for preclinical or prodromal AD. The established relationship between LOEU and AD suggests that patients with LOEU and consistent amyloid and tau biomarkers should be considered for epileptic preclinical or prodromal AD evaluation. To fully understand progression in LOEU, prospective studies that assess changes in imaging features and biomarkers over time are needed. Such studies will not only enhance our understanding of the underlying mechanisms but also improve accuracy in identifying of subgroups of LOEU at the greatest risk of AD.

Future research efforts should explore efficacy of amyloid lowering therapies or other targeted therapeutics in modifying the disease progression of both epilepsy and cognitive dysfunction in LOEU patients. By continuing to elucidate the interplay between LOEU and AD, potential treatment regimens may emerge that address both seizures and cognitive decline, thereby improving overall quality of life for individuals affected by LOEU.

## Author contributions

LH wrote the first draft of the manuscript. All authors contributed to manuscript conceptualization, and manuscript revision. All authors read and approved the submitted version.

## References

[1] Hauser WA Annegers JF Kurland LT. Incidence of epilepsy and unprovoked seizures in Rochester, Minnesota: 1935–1984. Epilepsia. (1993) 34:453–8. doi: 10.1111/j.1528-1157.1993.tb02586.x, PMID: 8504780

[2] Brodie MJ Elder AT Kwan P. Epilepsy in later life. Lancet Neurol. (2009) 8:1019–30. doi: 10.1016/S1474-4422(09)70240-6 19800848

[3] Lühdorf K Jensen LK Plesner AM. Etiology of seizures in the elderly. Epilepsia. (1986) 27:458–63. doi: 10.1111/j.1528-1157.1986.tb03567.x 3720706

[4] Tchalla AE Marin B Mignard C Bhalla D Tabailloux E Mignard D. Newly diagnosed epileptic seizures: focus on an elderly population on the French island of Réunion in the Southern Indian Ocean. Epilepsia. (2011) 52:2203–8. doi: 10.1111/j.1528-1167.2011.03320.x, PMID: 22091708

[5] Besocke AG Rosso B Cristiano E Valiensi SM García MC Gonorazky SE. Outcome of newly-diagnosed epilepsy in older patients. Epilepsy Behav. (2013) 27:29–35. doi: 10.1016/j.yebeh.2012.11.041, PMID: 23369763

[6] Costa C Parnetti L D’Amelio M Tozzi A Tantucci M Romigi A. Epilepsy, amyloid-β, and D1 dopamine receptors: a possible pathogenetic link? Neurobiol Aging. (2016) 48:161–71. doi: 10.1016/j.neurobiolaging.2016.08.025, PMID: 27701029

[7] Friedman D Honig LS Scarmeas N. Seizures and epilepsy in Alzheimer’s disease. CNS Neurosci Ther. (2012) 18:285–94. doi: 10.1111/j.1755-5949.2011.00251.x, PMID: 22070283 PMC3630499

[8] Vossel KA Beagle AJ Rabinovici GD Shu H Lee SE Naasan G. Seizures and epileptiform activity in the early stages of Alzheimer disease. JAMA Neurol. (2013) 70:1158–66. doi: 10.1001/jamaneurol.2013.136, PMID: 23835471 PMC4013391

[9] Vossel KA Tartaglia MC Nygaard HB Zeman AZ Miller BL. Epileptic activity in Alzheimer’s disease: causes and clinical relevance. Lancet Neurol. (2017) 16:311–22. doi: 10.1016/S1474-4422(17)30044-3, PMID: 28327340 PMC5973551

[10] Busche MA Chen X Henning HA Reichwald J Staufenbiel M Sakmann B. Critical role of soluble amyloid-β for early hippocampal hyperactivity in a mouse model of Alzheimer’s disease. Proc Natl Acad Sci U S A. (2012) 109:8740–5. doi: 10.1073/pnas.1206171109, PMID: 22592800 PMC3365221

[11] Paudel YN Angelopoulou E Jones NC O’Brien TJ Kwan P Piperi C. Tau related pathways as a connecting link between epilepsy and Alzheimer’s disease. ACS Chem Neurosci. (2019) 10:4199–212. doi: 10.1021/acschemneuro.9b00460, PMID: 31532186

[12] Minkeviciene R Rheims S Dobszay MB Zilberter M Hartikainen J Fülöp L. Amyloid beta-induced neuronal hyperexcitability triggers progressive epilepsy. J Neurosci. (2009) 29:3453–62. doi: 10.1523/JNEUROSCI.5215-08.2009, PMID: 19295151 PMC6665248

[13] Born HA Kim JY Savjani RR das P Dabaghian YA Guo Q. Genetic suppression of transgenic APP rescues hypersynchronous network activity in a mouse model of Alzeimer’s disease. J Neurosci. (2014) 34:3826–40. doi: 10.1523/JNEUROSCI.5171-13.2014, PMID: 24623762 PMC3951689

[14] Roberson ED Scearce-Levie K Palop JJ Yan F Cheng IH Wu T. Reducing endogenous tau ameliorates amyloid beta-induced deficits in an Alzheimer’s disease mouse model. Science. (2007) 316:750–4. doi: 10.1126/science.1141736, PMID: 17478722

[15] DeVos SL Goncharoff DK Chen G Kebodeaux CS Yamada K Stewart FR. Antisense reduction of tau in adult mice protects against seizures. J Neurosci. (2013) 33:12887–97. doi: 10.1523/JNEUROSCI.2107-13.2013, PMID: 23904623 PMC3728694

[16] Bellaver B Povala G Ferreira PCL Ferrari-Souza JP Leffa DT Lussier FZ. Astrocyte reactivity influences amyloid-β effects on tau pathology in preclinical Alzheimer’s disease. Nat Med. (2023) 29:1775–81. doi: 10.1038/s41591-023-02380-x, PMID: 37248300 PMC10353939

[17] Seifert G Carmignoto G Steinhäuser C. Astrocyte dysfunction in epilepsy. Brain Res Rev. (2010) 63:212–21. doi: 10.1016/j.brainresrev.2009.10.004 19883685

[18] Palop JJ Mucke L. Network abnormalities and interneuron dysfunction in Alzheimer disease. Nat Rev Neurosci. (2016) 17:777–92. doi: 10.1038/nrn.2016.141, PMID: 27829687 PMC8162106

[19] Alyenbaawi H Kanyo R Locskai LF Kamali-Jamil R DuVal MG Bai Q. Seizures are a druggable mechanistic link between TBI and subsequent tauopathy. elife. (2021) 10:e58744. doi: 10.7554/eLife.58744, PMID: 33527898 PMC7853719

[20] Hwang K Vaknalli RN Addo-Osafo K Vicente M Vossel K. Tauopathy and epilepsy comorbidities and underlying mechanisms. Front Aging Neurosci. (2022) 14:903973. doi: 10.3389/fnagi.2022.903973, PMID: 35923547 PMC9340804

[21] Sarkis RA Beers L Farah E al-Akaidi M Zhang Y Locascio JJ. The neurophysiology and seizure outcomes of late onset unexplained epilepsy. Clin Neurophysiol Off J Int Fed Clin Neurophysiol. (2020) 131:2667–72. doi: 10.1016/j.clinph.2020.08.014, PMID: 32957039 PMC7644268

[22] Kawakami O Koike Y Ando T Sugiura M Kato H Hiraga K. Incidence of dementia in patients with adult-onset epilepsy of unknown causes. J Neurol Sci. (2018) 395:71–6. doi: 10.1016/j.jns.2018.09.010, PMID: 30292966

[23] Hanby MF Al-Bachari S Makin F Vidyasagar R Parkes LM Emsley HCA. Structural and physiological MRI correlates of occult cerebrovascular disease in late-onset epilepsy. NeuroImage Clin. (2015) 9:128–33. doi: 10.1016/j.nicl.2015.07.016, PMID: 26413475 PMC4556750

[24] Krumholz A Wiebe S Gronseth GS Gloss DS Sanchez AM Kabir AA. Evidence-based guideline: management of an unprovoked first seizure in adults: report of the guideline development subcommittee of the American Academy of Neurology and the American Epilepsy Society. Neurology. (2015) 84:1705–13. doi: 10.1212/WNL.0000000000001487, PMID: 25901057 PMC4409581

[25] Lim JA Lee ST Moon J Jun JS Kim TJ Shin YW. Development of the clinical assessment scale in autoimmune encephalitis. Ann Neurol. (2019) 85:352–8. doi: 10.1002/ana.25421 30675918

[26] Bernasconi A Cendes F Theodore WH Gill RS Koepp MJ Hogan RE. Recommendations for the use of structural magnetic resonance imaging in the care of patients with epilepsy: a consensus report from the International League Against Epilepsy Neuroimaging Task Force. Epilepsia. (2019) 60:1054–68. doi: 10.1111/epi.15612, PMID: 31135062

[27] Zarow C Sitzer TE Chui HC. Understanding hippocampal sclerosis in the elderly: epidemiology, characterization, and diagnostic issues. Curr Neurol Neurosci Rep. (2008) 8:363–70. doi: 10.1007/s11910-008-0057-3, PMID: 18713571

[28] Amador-Ortiz C Lin WL Ahmed Z Personett D Davies P Duara R. TDP-43 immunoreactivity in hippocampal sclerosis and Alzheimer’s disease. Ann Neurol. (2007) 61:435–45. doi: 10.1002/ana.21154, PMID: 17469117 PMC2677204

[29] DeGiorgio CM Tomiyasu U Gott PS Treiman DM. Hippocampal pyramidal cell loss in human status epilepticus. Epilepsia. (1992) 33:23–7. doi: 10.1111/j.1528-1157.1992.tb02278.x 1733757

[30] Liguori C Costa C Franchini F Izzi F Spanetta M Cesarini EN. Cognitive performances in patients affected by late-onset epilepsy with unknown etiology: a 12-month follow-up study. Epilepsy Behav EB. (2019) 101:106592. doi: 10.1016/j.yebeh.2019.106592, PMID: 31726425

[31] Kaestner E Reyes A Chen A Rao J Macari AC Choi JY. Atrophy and cognitive profiles in older adults with temporal lobe epilepsy are similar to mild cognitive impairment. Brain J Neurol. (2021) 144:236–50. doi: 10.1093/brain/awaa397, PMID: 33279986 PMC7880670

[32] Cretin B Sellal F Philippi N Bousiges O di Bitonto L Martin-Hunyadi C. Epileptic prodromal Alzheimer’s disease, a retrospective study of 13 new cases: expanding the spectrum of Alzheimer’s disease to an epileptic variant? J Alzheimers Dis. (2016) 52:1125–33. doi: 10.3233/JAD-150096, PMID: 27104892

[33] Costa C Romoli M Liguori C Farotti L Eusebi P Bedetti C. Alzheimer’s disease and late-onset epilepsy of unknown origin: two faces of beta amyloid pathology. Neurobiol Aging. (2019) 73:61–7. doi: 10.1016/j.neurobiolaging.2018.09.006, PMID: 30317034

[34] Fernandes M Manfredi N Aluisantonio L Franchini F Chiaravalloti A Izzi F. Cognitive functioning, cerebrospinal fluid Alzheimer’s disease biomarkers and cerebral glucose metabolism in late-onset epilepsy of unknown aetiology: a prospective study. Eur J Neurosci. (2022) 56:5384–96. doi: 10.1111/ejn.15734, PMID: 35678770

[35] Nardi Cesarini E Babiloni C Salvadori N Farotti L del Percio C Pascarelli MT. Late-onset epilepsy with unknown etiology: a pilot study on neuropsychological profile, cerebrospinal fluid biomarkers, and quantitative EEG characteristics. Front Neurol. (2020) 11:199. doi: 10.3389/fneur.2020.00199, PMID: 32351438 PMC7174783

[36] Ophir K Ran B Felix B Amir G. Ten year cumulative incidence of dementia after late onset epilepsy of unknown etiology. J Clin Neurosci Off J Neurosurg Soc Australas. (2021) 86:247–51. doi: 10.1016/j.jocn.2021.01.030, PMID: 33775336

[37] Samson WN van Duijn CM Hop WC Hofman A. Clinical features and mortality in patients with early-onset Alzheimer’s disease. Eur Neurol. (1996) 36:103–6. doi: 10.1159/000117218 8654478

[38] DiFrancesco JC Tremolizzo L Polonia V Giussani G Bianchi E Franchi C. Adult-onset epilepsy in presymptomatic Alzheimer’s disease: a retrospective study. J Alzheimers Dis. (2017) 60:1267–74. doi: 10.3233/JAD-170392, PMID: 28968234

[39] Sarkis RA Dickerson BC Cole AJ Chemali ZN. Clinical and neurophysiologic characteristics of unprovoked seizures in patients diagnosed with dementia. J Neuropsychiatry Clin Neurosci. (2016) 28:56–61. doi: 10.1176/appi.neuropsych.15060143, PMID: 26404175

[40] Petersen RC Negash S. Mild cognitive impairment: an overview. CNS Spectr. (2008) 13:45–53. doi: 10.1017/s1092852900016151 18204414

[41] Keret O Hoang TD Xia F Rosen HJ Yaffe K. Association of late-onset unprovoked seizures of unknown etiology with the risk of developing dementia in older veterans. JAMA Neurol. (2020) 77:710–5. doi: 10.1001/jamaneurol.2020.0187, PMID: 32150220 PMC7063560

[42] Johnson EL Krauss GL Kucharska-Newton A Albert MS Brandt J Walker KA. Dementia in late-onset epilepsy: the atherosclerosis risk in communities study. Neurology. (2020) 95:e3248–56. doi: 10.1212/WNL.0000000000011080, PMID: 33097597 PMC7836657

[43] Wall J Knight J Emsley HCA. Late-onset epilepsy predicts stroke: systematic review and meta-analysis. Epilepsy Behav. (2021) 115:107634. doi: 10.1016/j.yebeh.2020.107634 33334717

[44] Johnson EL Krauss GL Lee AK Schneider ALC Dearborn JL Kucharska-Newton AM. Association between midlife risk factors and late-onset epilepsy: results from the atherosclerosis risk in communities study. JAMA Neurol. (2018) 75:1375–82. doi: 10.1001/jamaneurol.2018.1935, PMID: 30039175 PMC6248112

[45] Huang L Fu C Li J Peng S. Late-onset epilepsy and the risk of dementia: a systematic review and meta-analysis. Aging Clin Exp Res. (2022) 34:1771–9. doi: 10.1007/s40520-022-02118-8, PMID: 35428922

[46] Sen A Capelli V Husain M. Cognition and dementia in older patients with epilepsy. Brain J Neurol. (2018) 141:1592–608. doi: 10.1093/brain/awy022, PMID: 29506031 PMC5972564

[47] Joutsa J Rinne JO Hermann B Karrasch M Anttinen A Shinnar S. Association between childhood-onset epilepsy and amyloid burden 5 decades later. JAMA Neurol. (2017) 74:583–90. doi: 10.1001/jamaneurol.2016.6091, PMID: 28346588 PMC5822199

[48] Beghi E Cornaggia C RESt-1 Group. Morbidity and accidents in patients with epilepsy: results of a European cohort study. Epilepsia. (2002) 43:1076–83. doi: 10.1046/j.1528-1157.2002.18701.x, PMID: 12199734

[49] Beghi E Beghi M. Epilepsy, antiepileptic drugs and dementia. Curr Opin Neurol. (2020) 33:191–7. doi: 10.1097/WCO.0000000000000802 32073437

[50] Aldenkamp AP De Krom M Reijs R. Newer antiepileptic drugs and cognitive issues. Epilepsia. (2003) 44:21–9. doi: 10.1046/j.1528-1157.44.s4.3.x, PMID: 12823566

[51] Baxendale S. The impact of epilepsy surgery on cognition and behavior. Epilepsy Behav. (2008) 12:592–9. doi: 10.1016/j.yebeh.2007.12.015 18299253

[52] Breteler MM de Groot RR van Romunde LK Hofman A. Risk of dementia in patients with Parkinson’s disease, epilepsy, and severe head trauma: a register-based follow-up study. Am J Epidemiol. (1995) 142:1300–5. doi: 10.1093/oxfordjournals.aje.a117597, PMID: 7503050

[53] Liu CC Kanekiyo T Xu H Bu G. Apolipoprotein E and Alzheimer disease: risk, mechanisms, and therapy. Nat Rev Neurol. (2013) 9:106–18. doi: 10.1038/nrneurol.2012.263, PMID: 23296339 PMC3726719

[54] Fang Y Si X Wang J Chen Y Liu Y Yan Y. Alzheimer disease and epilepsy: a Mendelian randomization study. Neurology. (2023) 101:e399–e409. doi: 10.1212/WNL.0000000000207423 37225432 PMC10435057

[55] Blennow K Zetterberg H. Biomarkers for Alzheimer’s disease: current status and prospects for the future. J Intern Med. (2018) 284:643–63. doi: 10.1111/joim.12816, PMID: 30051512

[56] Jack CR Bennett DA Blennow K Carrillo MC Feldman HH Frisoni GB. A/T/N: an unbiased descriptive classification scheme for Alzheimer disease biomarkers. Neurology. (2016) 87:539–47. doi: 10.1212/WNL.0000000000002923, PMID: 27371494 PMC4970664

[57] Nagino N Kubota Y Nakamoto H Miyao S Kodama T Ito S. Non-lesional late-onset epilepsy in the elderly Japanese patients: presenting characteristics and seizure outcomes with regard to comorbid dementia. J Clin Neurosci. (2022) 103:100–6. doi: 10.1016/j.jocn.2022.05.003, PMID: 35868225

[58] Johnson EL Krauss GL Lee AK Schneider ALC Kucharska-Newton AM Huang J. Association between white matter hyperintensities, cortical volumes, and late-onset epilepsy. Neurology. (2019) 92:e988–95. doi: 10.1212/WNL.0000000000007010, PMID: 30804067 PMC6404466

[59] DiFrancesco JC Isella V Licciardo D Crivellaro C Musarra M Guerra L. Temporal lobe dysfunction in late-onset epilepsy of unknown origin. Epilepsy Behav. (2021) 117:107839. doi: 10.1016/j.yebeh.2021.107839, PMID: 33611099

[60] Sarkis RA Gale SA Yang HS Lam AD Singhal T Cicero S. Utility of amyloid positron emission tomography imaging in older adults with epilepsy and cognitive decline. Am J Alzheimers Dis Other Dement. (2023) 38:153331752311600. doi: 10.1177/15333175231160005, PMID: 36892007 PMC10580726

[61] Mackenzie IR Miller LA. Senile plaques in temporal lobe epilepsy. Acta Neuropathol (Berl). (1994) 87:504–10. doi: 10.1007/BF00294177, PMID: 8059603

[62] Sheng JG Boop FA Mrak RE Griffin WS. Increased neuronal beta-amyloid precursor protein expression in human temporal lobe epilepsy: association with interleukin-1 alpha immunoreactivity. J Neurochem. (1994) 63:1872–9. doi: 10.1046/j.1471-4159.1994.63051872.x, PMID: 7931344 PMC3833617

[63] Tai XY Koepp M Duncan JS Fox N Thompson P Baxendale S. Hyperphosphorylated tau in patients with refractory epilepsy correlates with cognitive decline: a study of temporal lobe resections. Brain J Neurol. (2016) 139:2441–55. doi: 10.1093/brain/aww187, PMID: 27497924 PMC5926008

[64] Thom M Liu JYW Thompson P Phadke R Narkiewicz M Martinian L. Neurofibrillary tangle pathology and Braak staging in chronic epilepsy in relation to traumatic brain injury and hippocampal sclerosis: a post-mortem study. Brain J Neurol. (2011) 134:2969–81. doi: 10.1093/brain/awr209, PMID: 21903728 PMC3187539

[65] Roe CM Fagan AM Grant EA Hassenstab J Moulder KL Maue Dreyfus D. Amyloid imaging and CSF biomarkers in predicting cognitive impairment up to 7.5 years later. Neurology. (2013) 80:1784–91. doi: 10.1212/WNL.0b013e3182918ca6, PMID: 23576620 PMC3719431

[66] Morris JC Blennow K Froelich L Nordberg A Soininen H Waldemar G. Harmonized diagnostic criteria for Alzheimer’s disease: recommendations. J Intern Med. (2014) 275:204–13. doi: 10.1111/joim.12199, PMID: 24605805

[67] Romoli M Sen A Parnetti L Calabresi P Costa C. Amyloid-β: a potential link between epilepsy and cognitive decline. Nat Rev Neurol. (2021) 17:469–85. doi: 10.1038/s41582-021-00505-9, PMID: 34117482

[68] Crary JF Trojanowski JQ Schneider JA Abisambra JF Abner EL Alafuzoff I. Primary age-related tauopathy (PART): a common pathology associated with human aging. Acta Neuropathol (Berl). (2014) 128:755–66. doi: 10.1007/s00401-014-1349-0, PMID: 25348064 PMC4257842

[69] Therriault J Vermeiren M Servaes S Tissot C Ashton NJ Benedet AL. Association of phosphorylated tau biomarkers with amyloid positron emission tomography vs tau positron emission tomography. JAMA Neurol. (2023) 80:188–99. doi: 10.1001/jamaneurol.2022.4485, PMID: 36508198 PMC9856704

[70] Barthélemy NR Li Y Joseph-Mathurin N Gordon BA Hassenstab J Benzinger TLS. A soluble phosphorylated tau signature links tau, amyloid and the evolution of stages of dominantly inherited Alzheimer’s disease. Nat Med. (2020) 26:398–407. doi: 10.1038/s41591-020-0781-z, PMID: 32161412 PMC7309367

[71] Hinman JD Elahi F Chong D. Placental growth factor as a sensitive biomarker for vascular cognitive impairment. Alzheimers Dement. (2023) 19:3519–27. doi: 10.1002/alz.12974, PMID: 36815663 PMC10440207

[72] Janelidze S Zetterberg H Mattsson N Palmqvist S Vanderstichele H Lindberg O. CSF Aβ42/Aβ40 and Aβ42/Aβ38 ratios: better diagnostic markers of Alzheimer disease. Ann Clin Transl Neurol. (2016) 3:154–65. doi: 10.1002/acn3.274, PMID: 27042676 PMC4774260

[73] Preische O Schultz SA Apel A Kuhle J Kaeser SA Barro C. Serum neurofilament dynamics predicts neurodegeneration and clinical progression in presymptomatic Alzheimer’s disease. Nat Med. (2019) 25:277–83. doi: 10.1038/s41591-018-0304-3, PMID: 30664784 PMC6367005

[74] Giovannini G Bedin R Ferraro D Vaudano AE Mandrioli J Meletti S. Serum neurofilament light as biomarker of seizure-related neuronal injury in status epilepticus. Epilepsia. (2022) 63:e23–9. doi: 10.1111/epi.17132, PMID: 34806176 PMC9299158

[75] Ouédraogo O Rébillard RM Jamann H Mamane VH Clénet ML Daigneault A. Increased frequency of proinflammatory CD4 T cells and pathological levels of serum neurofilament light chain in adult drug-resistant epilepsy. Epilepsia. (2021) 62:176–89. doi: 10.1111/epi.16742, PMID: 33140401

[76] Nation DA Sweeney MD Montagne A Sagare AP D’Orazio LM Pachicano M. Blood-brain barrier breakdown is an early biomarker of human cognitive dysfunction. Nat Med. (2019) 25:270–6. doi: 10.1038/s41591-018-0297-y, PMID: 30643288 PMC6367058

[77] Tabaee Damavandi P Storti B Fabin N Bianchi E Ferrarese C DiFrancesco JC. Epilepsy in cerebral amyloid angiopathy: an observational retrospective study of a large population. Epilepsia. (2023) 64:500–10. doi: 10.1111/epi.17489, PMID: 36515439

[78] Liguori C Spanetta M Romoli M Placidi F Nardi Cesarini E Mercuri NB. Sleep disorders and late-onset epilepsy of unknown origin: understanding new trajectories to brain amyloidopathy. Mech Ageing Dev. (2021) 194:111434. doi: 10.1016/j.mad.2021.111434, PMID: 33444630

[79] Mendez MF. What is the relationship of traumatic brain injury to dementia? J Alzheimers Dis. (2017) 57:667–81. doi: 10.3233/JAD-161002 28269777

[80] Hickman LB Patel AB Dubey I Karimi AH Zhang X Janio EA. Self-reported severity and causes of traumatic brain injury in patients with epileptic or functional seizures. Neurol Clin Pract. (2022) 12:e189–98. doi: 10.1212/CPJ.0000000000200098, PMID: 36540138 PMC9757118

[81] Milikovsky DZ Ofer J Senatorov VV Friedman AR Prager O Sheintuch L. Paroxysmal slow cortical activity in Alzheimer’s disease and epilepsy is associated with blood-brain barrier dysfunction. Sci Transl Med. (2019) 11:eaaw8954. doi: 10.1126/scitranslmed.aaw8954 31801888

[82] Cummings J Apostolova L Rabinovici GD Atri A Aisen P Greenberg S. Lecanemab: Appropriate Use Recommendations. J Prev Alzheimers Dis. (2023) 10:362–77. doi: 10.14283/jpad.2023.30, PMID: 37357276 PMC10313141

[83] Salloway S Chalkias S Barkhof F Burkett P Barakos J Purcell D. Amyloid-related imaging abnormalities in 2 phase 3 studies evaluating aducanumab in patients with early Alzheimer disease. JAMA Neurol. (2022) 79:13–21. doi: 10.1001/jamaneurol.2021.4161, PMID: 34807243 PMC8609465

[84] Sims JR Zimmer JA Evans CD Lu M Ardayfio P Sparks JD. Donanemab in early symptomatic Alzheimer disease: the TRAILBLAZER-ALZ 2 randomized clinical trial. JAMA. (2023) 330:e2313239. doi: 10.1001/jama.2023.13239 PMC10352931 37459141

[85] Javed A Cohen B Detyniecki K Hirsch LJ Legge A Chen B. Rates and predictors of patient-reported cognitive side effects of antiepileptic drugs: an extended follow-up. Seizure. (2015) 29:34–40. doi: 10.1016/j.seizure.2015.03.013, PMID: 26076842

[86] Fleisher AS Truran D Mai JT Langbaum JBS Aisen PS Cummings JL. Chronic divalproex sodium use and brain atrophy in Alzheimer disease. Neurology. (2011) 77:1263–71. doi: 10.1212/WNL.0b013e318230a16c, PMID: 21917762 PMC3179645

[87] Vossel K Ranasinghe KG Beagle AJ la A Ah Pook K Castro M. Effect of levetiracetam on cognition in patients with Alzheimer disease with and without epileptiform activity: a randomized clinical trial. JAMA Neurol. (2021) 78:1345–54. doi: 10.1001/jamaneurol.2021.3310, PMID: 34570177 PMC8477304

[88] French JA Perucca E Sander JW Bergfeldt L Baulac M Auerbach DS. FDA safety warning on the cardiac effects of lamotrigine: an advisory from the Ad Hoc ILAE/AES Task Force. Epilepsia Open. (2021) 6:45–8. doi: 10.1002/epi4.12475, PMID: 33681647 PMC7918301

